# Dentists and Anæsthesia

**Published:** 1909-05-15

**Authors:** Frederick W. Collingwood

**Affiliations:** Late Anæsthetist, National Dental Hospital


### EDITOR'S LETTER-BOX.

### "DENTISTS AND ANÆSTHESIA."

*To the Editor of* THE HOSPITAL.

SIR,—Your article on "Dentists and Anæsthesia" was of great interest to me. In my opinion, taking into consideration the course of study through which the modern dentist undergoes, including physiology, etc., if dental students had special instruction given to them (in nitrous oxide administration) during the period of studentship, there would be no danger to the public, were there a special clause in the proposed Bill now before Parliament, permitting them to administer nitrous oxide gas, but not extending to chloroform or ether. A dental student so instructed, with his constant future association with cases of nitrous oxide anæsthesia, and clinical experience gained therefrom would be as efficient an administrator of nitrous oxide as the general practitioner of medicine. It must be admitted, however, that if this privilege was conceded under the said Bill, the tendency would be for dental surgeons to attempt the double *rôle* of anæsthetic administrator and operator in individual cases, which certainly is fraught with danger to the patient. If this contention is valid, then two persons being necessarily present at the extraction of a tooth under nitrous oxide, the question arises, should that second person necessarily be a qualified practitioner of medicine. In my opinion the administrator of the gas should be a person other than the operator, and either a practitioner of medicine or a qualified dental surgeon.

I am, yours faithfully,

FREDERICK W. COLLINGWOOD.

Late Anæsthetist, National Dental Hospital.

